# Development of an indirect competitive enzyme-linked immunosorbent assay applied to the *Botrytis cinerea *quantification in tissues of postharvest fruits

**DOI:** 10.1186/1471-2180-11-220

**Published:** 2011-10-04

**Authors:** Martín A Fernández-Baldo, Jorge G Fernández, Sirley V Pereira, Germán A Messina, Eloy Salinas, Julio Raba, María I Sanz Ferramola

**Affiliations:** 1 INQUISAL, Departamento de Química. Universidad Nacional de San Luis, CONICET. Chacabuco 917. D5700BWS. San Luis, Argentina

## Abstract

**Background:**

*Botrytis cinerea *is a phytopathogenic fungus responsible for the disease known as gray mold, which causes substantial losses of fruits at postharvest. This fungus is present often as latent infection and an apparently healthy fruit can deteriorate suddenly due to the development of this infection. For this reason, rapid and sensitive methods are necessary for its detection and quantification. This article describes the development of an indirect competitive enzyme-linked immunosorbent assay (ELISA) for quantification of *B. cinerea *in apple (Red Delicious), table grape (pink Moscatel), and pear (William's) tissues.

**Results:**

The method was based in the competition for the binding site of monoclonal antibodies between *B. cinerea *antigens present in fruit tissues and *B. cinerea *purified antigens immobilized by a crosslinking agent onto the surface of the microtiter plates. The method was validated considering parameters such as selectivity, linearity, precision, accuracy and sensibility. The calculated detection limit was 0.97 μg mL-1 *B. cinerea *antigens. The immobilized antigen was perfectly stable for at least 4 months assuring the reproducibility of the assay. The fungus was detected and quantified in any of the fruits tested when the rot was not visible yet. Results were compared with a DNA quantification method and these studies showed good correlation.

**Conclusions:**

The developed method allowed detects the presence of *B. cinerea *in asymptomatic fruits and provides the advantages of low cost, easy operation, and short analysis time determination for its possible application in the phytosanitary programs of the fruit industry worldwide.

## Background

*Botrytis cinerea *is a pathogen ascomycete, which causes gray mold on a large number of economically important agricultural and horticultural crops [1-4]. This ubiquitous fungal pathogen is present often as latent infection. Latency is generally defined as the period between infection and the appearance of visible symptoms and can in the case of *B. cinerea *be long and variable [5-8]. Consequently, an apparently healthy fruit can deteriorate suddenly due to the development of this latent infection [9,10].

Many synthetic fungicides are used as the principal mean of controlling this important postharvest disease [11]. However, the growing public concern over the health and environmental hazards associated with fungicide use in orchards, the development of fungicide resistant strains of *B. cinerea *[12], and the deregistration of some of the most effective fungicides [13], have generated a great interest in the development of alternative methods to control the postharvest disease caused by this fungal pathogen.

To prevent the indiscriminate use of fungicides, a sensitive and reliable method to early determination of the fungus in fruit tissues becomes crucial. The ability to detect latent infections in fruit tissues should prove useful not only for early disease management but also for identifying infected fruit in postharvest. In addition, the quantification of the pathogen is necessary for the application of alternative methods of control, such as biological control using antagonist microorganisms because the success of this method depend of the ratio antagonist/pathogen [14].

The detection of fungus in fruit includes classical methods such as isolation on selective media, which is useful but subject to limitations [15] due to many pathogens can be masked by overgrowth of faster growing fungi. Other methods, such as quantitative real-time polymerase chain reaction (Q-PCR), or reverse transcription polymerase chain reaction (RT-PCR) represent new tools for the detection of the pathogens by determination of their DNA/RNA [16-25]. Unfortunately these methods are expensive and not easy to perform routinely, because they require highly qualified personnel and need sophisticated instrumentation [26,27]. In addition, to methods mentioned previously, some direct enzyme-linked immunosorbent assays (ELISAs) using microtiter plates have been developed for the detection of *B. cinerea *in pear steam, grape juice, and plants [28-32], but at present has not been reported any validated method based in an indirect competitive immunoassay for detection and quantification of the mentioned fungus in tissues of fruits.

The aim of this study was the development and corroboration of a sensitive and specific ELISA for *B. cinerea *quantification in fruit post-harvest tissues such as apple (Red Delicious), table grape (pink Moscatel), and pear (William's). The determination of *B. cinerea *was based in an indirect competitive immunoassay that used purified *B. cinerea *antigens, which were immobilized on the surface of the microtiter plates by a crosslinking agent. The *B. cinerea *specific monoclonal antibodies (BC-12.CA4) were allowed to react immunologically with immobilized antigens and with *B. cinerea *antigens present in the fruit sample. These antigens compete for the binding site of antibodies. Those antibodies whose binding site reacted with the immobilized antigens were detected by a horseradish peroxidase (HRP) enzyme-labeled second antibodies specific to mouse IgG, using a substrate solution. The response colour obtained from the product of enzymatic reaction (P) was measured by an ELISA microplate reader at 490 nm and the colour signal was inversely proportional to the amount of *B. cinerea *antigens present in the fruit sample. The method was validated considering parameters such as selectivity, linearity, precision, accuracy, and sensibility. The results obtained were correlated with the damage produced in the infected fruits by the pathogen and with the DNA of *B. cinerea *that was recovered from the lesions.

## Results and discussion

### Preparation of antigens and samples

The preparation of purified antigen and samples included a treatment with liquid nitrogen with the aim of exposing the antigenic sites. In preliminary tests this step was not taken into account, and the resulting signal was very low. According Meyer et al, the monoclonal antibody, BC-12.CA4 recognizes an antigen, possibly a glycoprotein, with the antigenic binding site on L-rhamnose and the treatment with liquid nitrogen help to expose these sites in high quantities [29].

Purified antigens were immobilized on the surface of the microtiter plates by a crosslinking agent and were stable for at least 4 months.

### Quantitative test for the determination of B. cinerea

The fruit samples consisted in apples (Red Delicious), table grape (pink Moscatel), and pear (William's) without any postharvest treatment and were purchased from a local fruit market in San Luis City, Argentina

The method was applied for the determination of *B. cinerea *in 50 commercial fruit samples. All fruits were selected as much as possible homogeneous in maturity and size.

Because the developed method was based in a competition between *B. cinerea *purified antigens immobilized onto the surface of the microtiter plates, and *B. cinerea *antigens present in fruit tissues, the absorbance at 490 nm was inversely proportional to the amount of the *B. cinerea *antigen present in the fruit sample.

**Figure 1:**
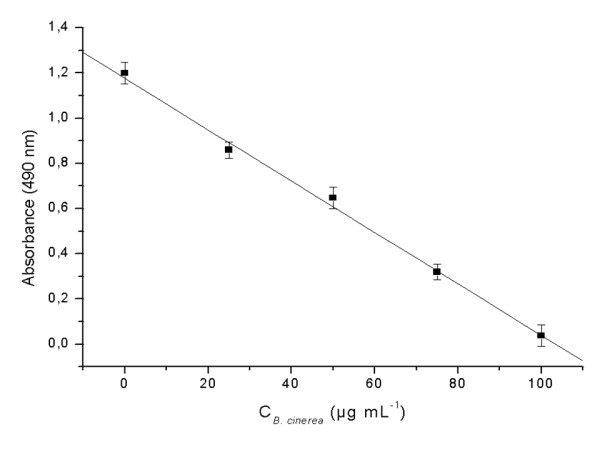
**Standard curve for the indirect competitive ELISA made with purified antigens of *B. cinerea *covering a range of antigen concentration between 0 and 100 μg mL^-1 ^**. Each value is based on five determinations. The error values represent the standard deviation.

A standard curve for the immunoassay procedure was carried out following our protocol with a series of purified antigens that covered a relevant range of concentration (0-100 μg mL^-1 ^antigen) (Figure 1). The linear regression equation was A = 1.18 - 0.01 * C_*B. cinerea*_, with the linear regression coefficient r = 0.998 and a detection limit (DL) of 0.97 μg mL^-1^. The DL was considered to be the concentration that gives a signal three times the standard deviation (SD) of the blank.

The coefficient of variation (CV) for the determination of 25 μg mL^-1 ^*B. cinerea *was below 4% (six replicates).

The precision of the ELISA assay was checked with control solutions of 5, 25 and 75 μg mL^-1 ^*B. cinerea *purified antigens concentrations. The within-assay precision was tested with 5 measurements in the same run for each sample. These series of analyses were repeated for three consecutive days in order to estimate the between-assay precision. The results obtained are presented in Table 1. The *B. cinerea *immunoassay showed good precision; the CV within-assay values were below 4% and the between-assay values were below 7%.

**Table 1:** Within-assay precision (five measurements in the same run for each control) and between-assay precision (five measurements for each control, repeated for three consecutive days).

^a ^Control solution	Within-assay		Between-assay	
	
	Mean	CV %	Mean	CV %
5 μg mL^-1^	5.27	3.51	5.87	4.56
25 μg mL^-1^	24.56	2.87	25.30	5.80
75 μg mL^-1^	75.92	3.15	74.17	6.58

^a ^μg mL^-1 ^*B. cinerea *antigen

The correlations between the lesion diameters of the fruit samples and the amount of *B. cinerea *antigen detected by the proposed method from infected fruit extracts samples obtained at 4, 7, and 10 d of incubation (25°C), respectively, are presented in Table 2. These results showed a correlation between the damage level and the amount of fungus present in the fruit samples. *B. cinerea *was detected even when the fruit rot was not visible yet but perhaps it had begun to germinate (about 4 days after inoculation and incubation of the fruit samples). Tests in which the fruit samples were infected using different conidia suspensions of *B. cinerea *were also made: 1 × 10^4^, 1 × 10^5^, and 1 × 10^6 ^conidia mL^-1^, respectively. Absorbance measured after 4 d of incubation (25°C) did not show significant differences (data not shown), because the method only detect germ tubes in the precise moment they appear, and the quantity of germinated conidia does not always depend of the quantity of inoculated conidia.

**Table 2:** Correlation between the lesion diameters of the fruit samples, the amount of *B. cinerea *antigen determinated by the ELISA developed and the DNA of *B. cinerea *quantified from infected fruit extracts samples obtained at 4, 7, and 10 days of incubation (25°C), respectively.

Fruit samples	Days of incubation	^**b**^**Lesion diameters (mm/rot)**	^c ^*B. cinerea *antigen (μg mL^-1^)	^c ^*DNA- B. cinerea* (μg mL^-1^)
Apples (Red-delicious)	^**a **^Control	uninfected	not detected	not detected
	4	not visible	10.53 ± 0.48	10.22 ± 0.53
	7	20.11 ± 0.54	40.67 ± 0.37	38.75 ± 0.41
	10	50.09 ± 4.49	69.08 ± 0.43	71.19 ± 0.37
Table grapes (pink Moscatel)	^**a **^Control	uninfected	not detected	not detected
	4	not visible	14.26 ± 0.51	13.86 ± 0.54
	7	3.69 ± 0.52	49.03 ± 0.46	51.99 ± 0.42
	10	5.35 ± 0.14	77.18 ± 0.36	75.84 ± 0.41
Pears (William's)	^**a **^Control	uninfected	not detected	not detected
	4	not visible	11.29 ± 0.47	12.76 ± 0.51
	7	15.13 ± 1.23	41.78 ± 0.55	41.44 ± 0.48
	10	38.98 ± 1.67	70.84 ± 0.49	72.39 ± 0.52

**^a ^**Negative control (uninfected fruits).

**^b ^**Diameters of the lesion measured in the fruit samples at 4, 7 and 10 days of incubation (25°C) respectively.

**^b, c ^**X (μg mL^-1^), mean ± SD, standard deviation.

**Figure 2:**
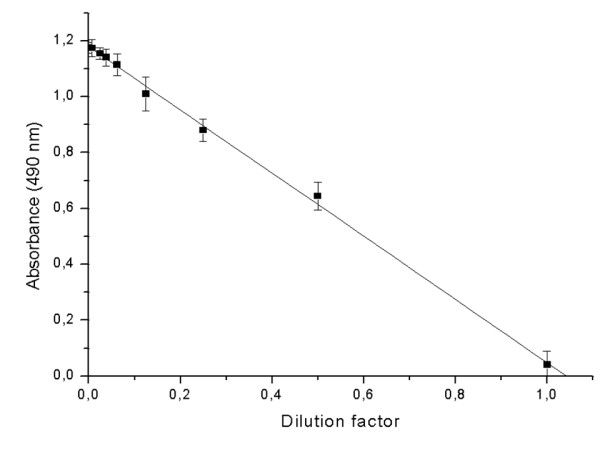
**Dilution test using a control solution of 100 μg mL^-1 ^*B. cinerea *purified antigen**. Dilutions were made with 0.01 M PBS, pH 7.2. Each value is based on five determinations. The error values represent the standard deviation.

The accuracy was tested with dilution and recovery tests. A dilution test was performed with a control solution of 100 μg mL^-1 ^*B. cinerea *purified antigens concentration in 0.01 M PBS, pH 7.2 (Figure 2).

Reproducibility assays were made using a repetitive standard (n = 6) of 25 μg mL^-1 ^*B. cinerea *(Table 3).

**Table 3:** Reproducibility assays using repetitive standards (n = 6) of 25 μg mL^-1 ^*B. cinerea *antigen concentration.

**Standards of 25 μg mL**^**-1 **^***B. cinerea *antigen**	Proposed method (μg mL^-1^)
1	25.60
2	25.20
3	24.16
4	25.15
5	24.98
6	24.49

*^a ^*X ± SD	24.93 ± 0.52

*^a ^*X (μg mL^-1^), mean ± SD, standard deviation.

The results obtained showed that the method developed had a lower Detection Limit and a shorter total assay time, than the non-competitive ELISA previously reported, and provided a wider dynamic range [28-32]. In addition, this method ELISA was developed for the quantification of *B. cinerea *in a complex matrix such as fruit tissues (apples, table grapes and pears samples).

### Cross-reactivity studies with fungi isolated from fruits

The cross reactivity test of the monoclonal antibody for *B. cinerea *with the fungi frequently isolated from fruits (apples, table grapes and pears) resulted in no cross-reactions, indicating that the antibody was specific to *B. cinerea*. The phytopathogens assayed were *Penicillium expansum *CEREMIC 151-2002, *Aspergillus niger *NRRL 1419, *Aspergillus ochraceus *NRRL 3174, *Alternaria sp*. NRRL 6410, *Rhizopus sp*. NRRL 695. In all cases absorbance read at 490 nm corresponded to maximum value indicating that the sample did not contain competitive antigens. We confirmed findings obtained by Meyer et al. [29], that BC-12.CA4 is highly selective to *B. cinerea*.

### Comparison of the proposed method with a DNA quantification method

The method developed was compared with a DNA quantification method [33] for *B. cinerea *in 45 fruit samples (15 fruit samples of each kind: apple, table grape and pear). Concentrations of DNA were detected spectrophotometrically by measuring absorbance changes at 260 nm showed good integrity by the high molecular weight bands on electrophoresis (data not shown). The analysis was carried out with the extracts of fruits at 4, 7, and 10 d of incubation (25°C), simultaneously with ELISA assay. The results obtained indicate a good correspondence between the two methods (Table 2). These results suggest that the sensitivity reached for this procedure allow determining very low level of *B. cinerea *antigens in apparently healthy fruit that can deteriorate suddenly due to the development of latent or quiescent infection into visible disease. Also, the DNA quantified by the method developed by González et al. [33] from uninfected and infected fruit extracts samples was amplified by PCR, with the purpose of verify if the same correspond to specific DNA of *B. cinerea *[34].

**Figure 3:**
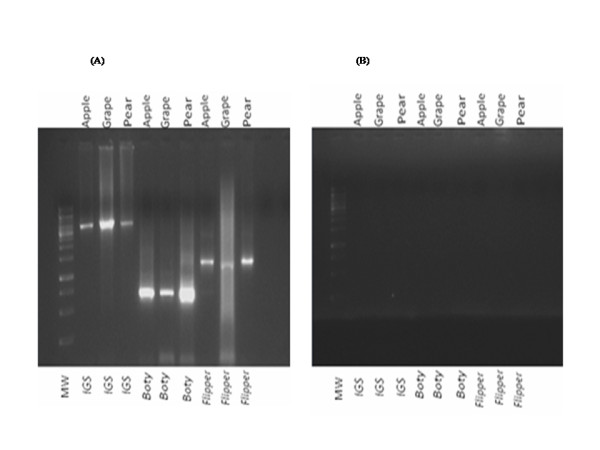
**Gels show one sample of each kind of infected fruit extract with conidial suspensions (1 × 10^5 ^spores mL^-1^) and a control per each kind of uninfected fruit extract sample**. ***(A) *PCR product analysis of infected fruit extracts samples**. Lane 1: standard molecular weight marker (MW). Lanes 2, 3 and 4: molecular marker IGS (ribosomal intergenic spacer). Lanes 5, 6 and 7: *Boty *transposable element. Lanes 8, 9 and 10: *Flipper *transposable element. ***(B) *PCR product analysis of uninfected fruit extracts samples**. Lane 1: standard molecular weight marker (MW). Lanes 2, 3, 4, 5, 6, 7, 8, 9 and 10: not observed any bands, indicating clearly the absence of *B. cinerea*.

The Figure 3A shows the DNA-*B. cinerea *from infected fruit extracts samples (apples, table grapes and pears respectively). The bands observed in the lane 1 correspond to a standard of molecular weight marker (MW); in the lanes 2, 3 and 4 correspond to a molecular marker (IGS) for each fruit extracts; in the lanes 5, 6 and 7 correspond to the *Boty *transposable element for each fruit extract and in the lanes 8, 9 and 10 correspond to the *Flipper *transposable element for each fruit extract. The Figure 3B shows control extracts made from uninfected fruits. There, only were observed bands in the lane 1 which correspond to a standard of molecular weight marker (MW) indicating clearly the absence of *B. cinerea*.

The presence of both transposable elements (*Boty *and *Flipper*) indicates that *B. cinerea *can be molecularly characterized as subpoblation transposa-type [35,36].

## Conclusions

In the present study, a specific and sensitive indirect competitive ELISA for the quantification of *B. cinerea *in commercial apple, table grape and pear samples was developed and validated. This inexpensive and simplified method can be applied for 96 fruit samples, per each microtiter plate with a total time for the assay of 35 min. Preparations of immobilized antigen on surface microtiter plates were perfectly stable for at least 4 months assuring the reproducibility of the assay. This is one important advantage for the possible commercialization of the developed ELISA.

The results obtained suggest that the sensitivity reached for this procedure allows determining very low levels of *B. cinerea *antigens in apparently healthy fruits. Also, the validation procedures showed that the method developed was reliable and accurate and that was possible to correlate the quantities of *B. cinerea *antigens with DNA of *B. cinerea *present in fruit tissues. In addition, the immunological reaction between monoclonal antibodies for *B. cinerea *and antigens from others fungi, frequently isolated from fruits resulted in no cross-reactions.

In conclusion, this method promises to be particularly useful in the analysis of symptomless fruits, either to locate latent infections, avoiding thus, conventional culturing techniques, which are not only time-consuming, but also are not able to give a quantitative result.

## Methods

### Reagents and Solutions

All reagents used were of analytical reagent grade. The monoclonal antibody for *B. cinerea *(BC-12.CA4) and the secondary antibody-enzyme conjugate (anti-mouse polyvalent immunoglobulins peroxidase conjugate) were obtained from *ADGEN *diagnostics (Auchincruive, Scotland) and Sigma Chemical (St. Louis, MO, USA) respectively. Glutaraldehyde (25% aqueous solution), hydrogen peroxide (H_2_O_2_), sodium clorure (NaCl) and sulfuric acid (H_2_SO_4_) were purchased from Merck (Darmstadt, Germany). Bovine serum albumin (BSA), Horseradish peroxidase (HRP), orthophenylenediamine (OPD) and Tween 20 were purchased from Sigma-Aldrich (St. Louis, MO, USA). All other reagents employed were of analytical grade and were used without further purification. Aqueous solutions were prepared using purified water from a Milli-Q-system. ELISA plate (Costar 3590, high binding polystyrene, 96 wells assay plate) was purchased from Costar (Corning, Massachusetts, USA).

### Intrumentation

All solutions and reagents were conditioned to 37°C before the experiment, using a laboratory water bath Vicking Mason Ii (Vicking SRL, Argentina).

All pH measurements were made with an Orion Expandable Ion Analyzer (model EA 940, Orion Research, Cambridge, MA, USA) equipped with a glass combination electrode (Orion Research).

Absorbance was measured with an automatic ELISA reader (Bio-Rad 3550-UV Microplate Reader, Japan) and Beckman DU 520 General UV/vis spectrophotometer (USA).

All polymerase chain reactions (PCR) were carried out on the PCR Thermocycler (BIO-RAD, USA).

Microscopic studies were carried out on the Olympus CH 30 (Spectra services, N.Y., USA).

### PCR assays

The primers used for PCR assays were: ribosomal region 18S (IGS spacer) 5'-ATGAGCCATTCGCAGTTTC-3' (GenBank Accession no: J01353). To determine the transposable elements status of each isolate, whether they were of *vacuma *or *transposa *type, we focused on the detection of *Flipper *with the primers F-300 5'GCACAAAACCTACAGAAGA-3' (GenBank Accession no: U74294) and the detection of *Boty *with the two primers *B*-R 5'-TAACCTTGTCTTTGCTCATC-3 and *B*-L 5'-CCCAATTT-ATTCAATGTCAG-3'. (GenBank Accession no: X81790 and X81791).

Each reaction was performed with: 6 μL of primers, 2.5 μL of dNTP, 2.5 μL of DNA, 2.5 μL of Mg^+2^, and 0.5 μL of Taq polymerase in a total volume of 50 μL.

PCR amplification conditions were: an initial denaturing step of 94°C by 4 s; 35 cycles of 94°C by 1 s, 60°C by 1 s and 72°C by 210 s; and a final elongation step of 4 s at 72°C (Muñoz et al., 2008). The products were analyzed on agarose gel 2%, stained with ethidium bromide and then observed under UV light (Figure 3).

### Preparation of the B. cinerea antigens

The purified *B. cinerea *antigens were prepared following the same procedure as a previous work [37].

*B. cinerea *Pers.: Fr (BNM 0527) was used in this study. The strain is deposited in the National Bank of Microorganisms (WDCM938) of the Facultad de Agronomia, Universidad de Buenos Aires (FAUBA). The isolates were maintained on potato dextrose agar (PDA) at 4°C.

To induce the mycelial production, *B. cinerea *was grown on PDA for 8-12 days at 21 ± 2°C. After this incubation period, the mycelium was removed, frozen in liquid nitrogen, blended in a Waring^® ^blender, and freeze-dried to obtain a fine powder. Then, the fine powder was suspended in 0.01 M phosphate buffer (PBS, pH 7.2) and centrifuged at 1000 × g for 10 min. The supernatant, which contained the antigen, was stored in 0.01 M PBS, pH 7.2, at -20°C between uses. In this study, the concentration of antigen was expressed as *Botrytis *antigen units (B-AgU), which was equivalent to μg mL^-1 ^PBS extracts of freeze-dried fungal mycelium [29].

To induce the conidial production, *B. cinerea *was grown on PDA at 21 ± 2°C until apparition of the mycelium, then the cultures were maintained at 15°C during a week. The conidia were harvested and suspended in 10 mL of sterile 0.01 M PBS (pH 7.2) containing 0.05% (v/v) Tween 80.

Finally, the concentration of spore suspension was determined with a Neubauer chamber and adjusted with in 0.01 M PBS (pH 7.2) to 1 × 10^5 ^conidia mL^-1^. This conidia suspension was used to infect the fruit samples.

### Immobilization of purified antigen of B. cinerea on surface microtiter plates

As the first step of the immobilization of purified antigen procedure, the microtiter plates were coated and incubated 4 h at room temperature in a moist chamber, with 100 μL per well of an aqueous solution of 5% (w/w) glutaraldehyde at pH 10 (0.20 M sodium carbonate buffer) diluted 1:2 in 0.1 M PBS (pH 5). After washing twice with 0.1 M PBS (pH 5), 100 μL per well of antigens preparation (10 μg mL^-1 ^0.01 M PBS, pH 7.2) were coupled to the residual aldehyde groups for 3 h at 37°C. Later, two washes with 0.9% NaCl and three washes with 0.01 M PBS (pH 7.2) were carried out. After these wash steps, the surface of each well was blocked with 200 μL of 1.5% BSA in 0.01 M PBS (pH 7.2) for 1 h at 37°C. The immobilized antigen was washed three times with PBST (0.8% NaCl, 0.11% Na_2_HPO_4_, 0.02% KH_2_PO_4_, 0.02% KCl, 0.05% Tween 20, pH 7.2).

Finally allowed to dry 5 min at room temperature and stored at -20°C until use. Preparations of immobilized antigen were perfectly stable for at least 4 months.

### Indirect competitive ELISA for the B. cinerea quantification

#### Preparation of infected fruit extracts samples

The preparation of infected fruit extracts samples was carried out according to the procedure described in our previous article [37].

In a first step, the fruit samples were infected using a spore suspension (1 × 10^5 ^conidia mL^-1^). Apples, pears, and table grapes were wounded using a punch. The wound size of apples and pears was 3 mm × 3 mm × 3 mm, whereas the one of table grapes was 1 mm × 1 mm × 1 mm. After that, 20 μL of the conidia suspension was put into each wound. Then, the fruits were kept at 25°C and the evaluations of rot incidence and lesion diameters were made over 10 days. Ten fruits were used for each assay with three wounds each. Each experiment was repeated three times.

In a second step, fruit tissues infected and uninfected were removed and were ground to a fine powder in liquid N_2_.

Finally, the infected fruit extracts samples were prepared by adding 0.1 g of powdered fruit tissue into 0.9 mL of 0.01 M PBS (pH 7.2) and vortexed for 1 min to obtain a homogeneous suspension, which was used in the immunological assay.

### Description of the immunological test

**Figure 4:**
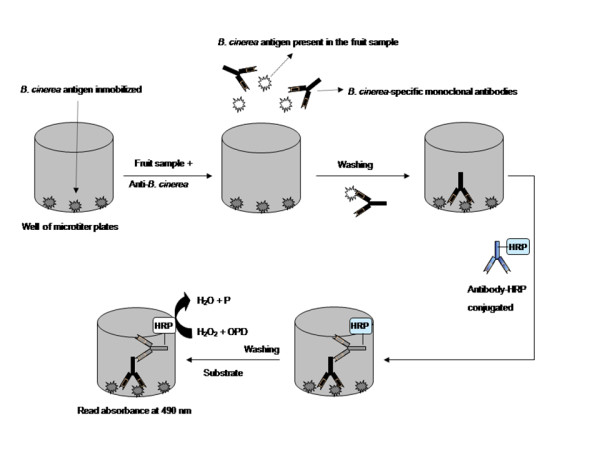
**Scheme of the indirect competitive immunoassay**.

Before starting the assay the microtiter plate with immobilized antigens was carried at room temperature for 5 min. After, 25 μL of fruit extracts samples and 25 μL of the monoclonal antibody IgG mouse anti-*B. cinerea *(15 μg mL^-1 ^in 0.01 M PBS, pH 7.2) were added to wells and incubated for 10 min at 37°C. In this step, *B. cinerea *present in the fruit sample was allowed to compete by the specific monoclonal antibody with the immobilized purified *B. cinerea *antigens on surface of microtiter plates (Figure 4). After that, the plates were washed three times with PBST. Then, 50 μL of the anti-mouse IgG-HRP conjugate (diluted 0.75:1500 in 0.01 M PBS, pH 7.2) were added and incubated for 5 min at 37°C. The plate was washed again three times with PBST and finally, 50 μL of substrate solution (OPD 4 mg/5 mL; PCB 0.1 M phosphate citrate, 10 μL H_2_O_2_) per well, were incorporated, and incubated for 3 min at room temperature. After 3 min, the reaction was stopped with 50 μL of 4 N H_2_SO_4_. Absorbance values were determined using a microplate reader at 490 nm.

The stock solution of substrate was prepared freshly before the experiment and stored in the darkness for the duration of the experiment.

### Cross-reactivity studies with fungi isolated from fruits

For the cross reaction study, the phytopathogenic fungi most common in Argentina were assayed. *Penicillium expansum *CEREMIC 151-2002, *Aspergillus niger *NRRL 1419, *Aspergillus ochraceus *NRRL 3174, *Alternaria sp*. NRRL 6410, *Rhizopus sp*. NRRL 695) were isolated from fruits (apples, table grapes and pears). Single spore cultures were incubated on PDA for 7 to 10 days at 21 ± 2°C. Water-soluble surface antigens were removed from plate cultures by flooding plates with 5 mL of 0.01 M PBS, pH 7.2. Solutions obtained previously were transferred to 1.5-mL eppendorf tubes and centrifuged to remove particulate materials. The supernatant was diluted 1:5 with 0.01 M PBS, pH 7.2 and used as described our method above, except that the 25 μL of fruit extracts were replaced for 25 μl of the diluted supernatant (phytopathogenic fungi isolated from fruits). Finally, the absorbance was measured by ELISA microplate reader at 490 nm.

## Authors' contributions

MFB participated in the design of the study, performed experiments and drafted the manuscript. JF carried out the molecular genetic studies. SP and GM contributed to coordinate the study. ES helped in microbiological assays and in the obtention of antigen. JR helped to draft the manuscript and critically revised the manuscript. MSF participated in the study conception and coordination, provided guidance during all parts of the work, and helped to draft the manuscript. All authors read and approved the final version of the manuscript.
